# Supplementary material for: Global short-term forecasting of COVID-19 cases
Source: Sci Rep. 2021 Apr 6;11:7555. doi: 10.1038/s41598-021-87230-x (PMC8024267; doi:10.1038/s41598-021-87230-x)
Supplement: Supplementary file 1 — Supplementary Information 1. [file 41598_2021_87230_MOESM1_ESM.pdf]

Global Short-Term Forecasting of Covid-19 Cases

Thiago de Paula Oliveira[1,+] and Rafael de Andrade Moral[2,+,*]

[1]The Roslin Institute and Royal (Dick) School of Veterinary Studies, University of Edinburgh, Roslin, UK
[2]Maynooth University, Department of Mathematics and Statistics and Hamilton Institute, Maynooth, W23 F2H6, Ireland
[*]rafael.deandrademoral@mu.ie
[+]these authors contributed equally to this work

Supplementary Material

Table S1 displays parameter estimates for the model fitted to the data up to 25-Nov-2020. Figures S1–S4 depict the four additional forecast validation procedures we have carried out. Figure S5 displays density plots for the sampled posterior distributions for each model parameter. Figures S6–S14 display estimated autoregressive components $\hat{\gamma}_{it}$ for each country for the model fitted to the data up to 25-Nov-2020.

Parameter	Estimate	95% CI [lower; upper]
β_0	0.9994	$[0.9984, 1.0004]$
β_1	-0.0421	$[-0.0541, -0.0298]$
β_2	0.0420	$[0.0306, 0.0542]$
σ_{b_0}	0.0051	$[0.0045, 0.0057]$
σ_{b_1}	0.0188	$[0.0126, 0.0272]$
σ_{b_2}	0.0186	$[0.0125, 0.0269]$
σ_η	0.3320	$[0.3260, 0.3378]$
π	0.1255	$[0.1210, 0.1303]$
σ_ω	4.9424	$[4.7486, 5.1597]$
ψ	0.0094	$[0.0067, 0.0124]$

Table S1. Parameter estimates and associated 95% credible intervals (CI) for the fitted autoregressive hierarchical state-space negative binomial model. β_0, β_1 and β_2 are the fixed effects associated with the time orthogonal polynomials of order 0, 1 and 2, respectively; σ_{b_0}, σ_{b_1} and σ_{b_2} are the standard deviations of the random effects associated with the time orthogonal polynomials of order 0, 1 and 2, respectively; σ_η is the standard deviation of the random autoregressive process; π is the probability of an observation being an outlier and σ_ω is the standard deviation of the mixture component ω_{it}; ψ is the dispersion parameter of the negative binomial distribution.

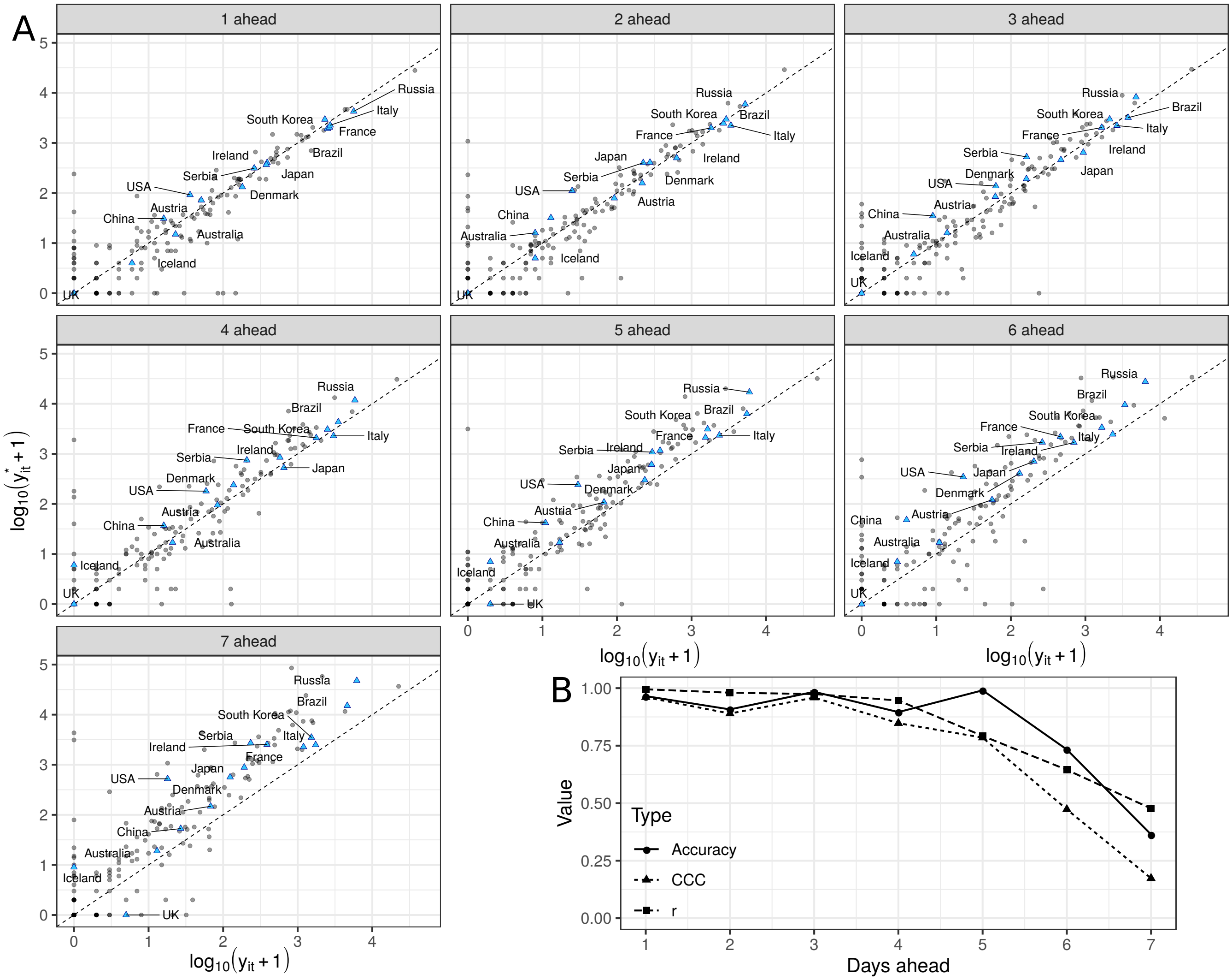

Figure S1. **A** Logarithm of the observed y_{it} versus the forecasted daily number of cases y^*_{it} for each country, for up to seven days ahead, where each day ahead constitutes one panel. The forecasts were obtained from the autoregressive state-space hierarchical negative binomial model, fitted using data up to 29-April-2020. The first day ahead corresponds to 30-April-2020, and the seventh to 06-May-2020. Each dot represents a country, and the sixteen countries shown in Figure 2 are represented by blue triangles. We add 1 to the values before taking the logarithm. **B** Observed accuracy, concordance correlation coefficient (CCC) and Pearson correlation (r) between observed (y_{it}) and forecasted (y^*_{it}) values for each of the days ahead of 29-April-2020.

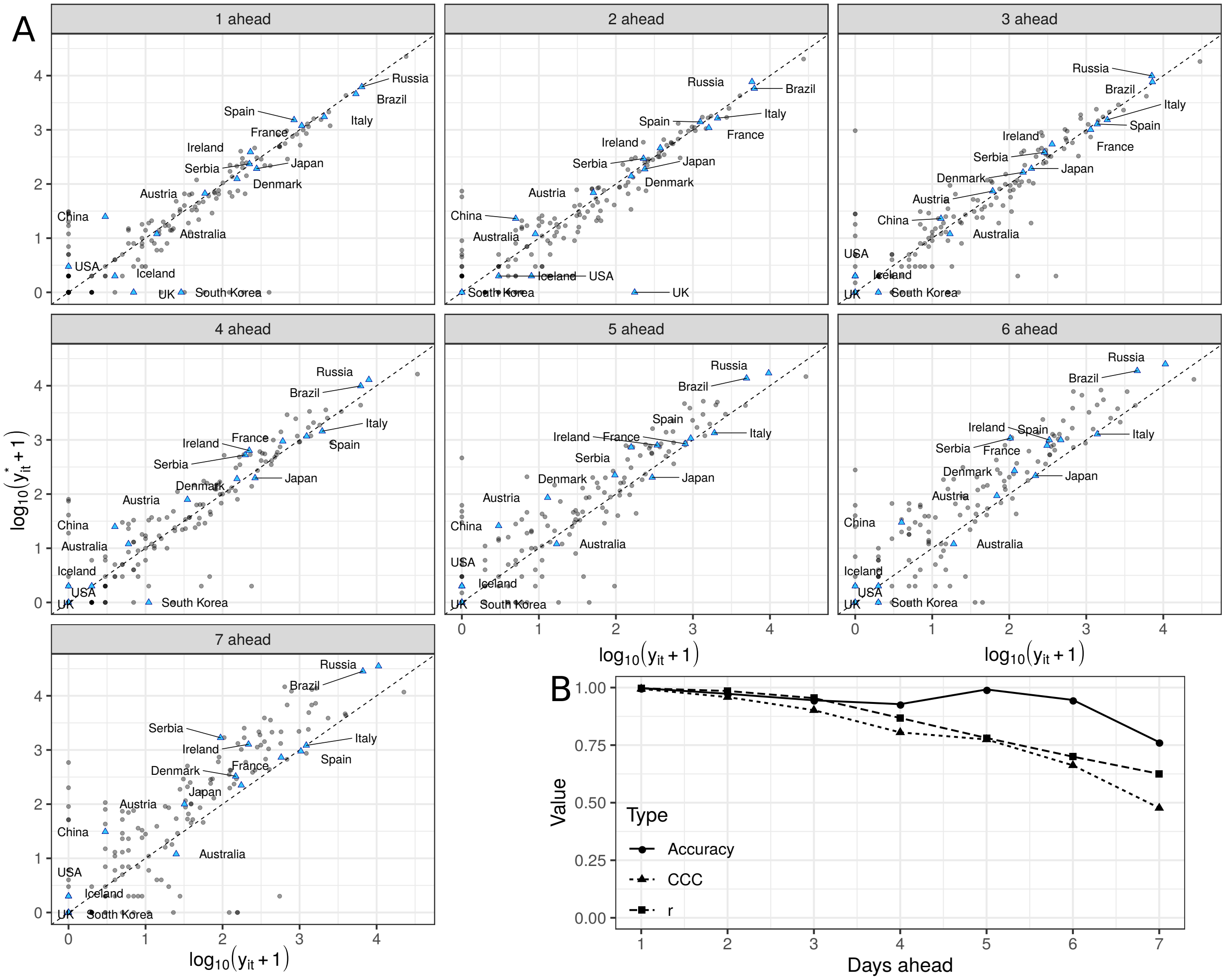

Figure S2. **A** Logarithm of the observed y_{it} versus the forecasted daily number of cases y^*_{it} for each country, for up to seven days ahead, where each day ahead constitutes one panel. The forecasts were obtained from the autoregressive state-space hierarchical negative binomial model, fitted using data up to 06-May-2020. The first day ahead corresponds to 07-May-2020, and the seventh to 13-May-2020. Each dot represents a country, and the sixteen countries shown in Figure 2 are represented by blue triangles. We add 1 to the values before taking the logarithm. **B** Observed accuracy, concordance correlation coefficient (CCC) and Pearson correlation (r) between observed (y_{it}) and forecasted (y^*_{it}) values for each of the days ahead of 06-May-2020.

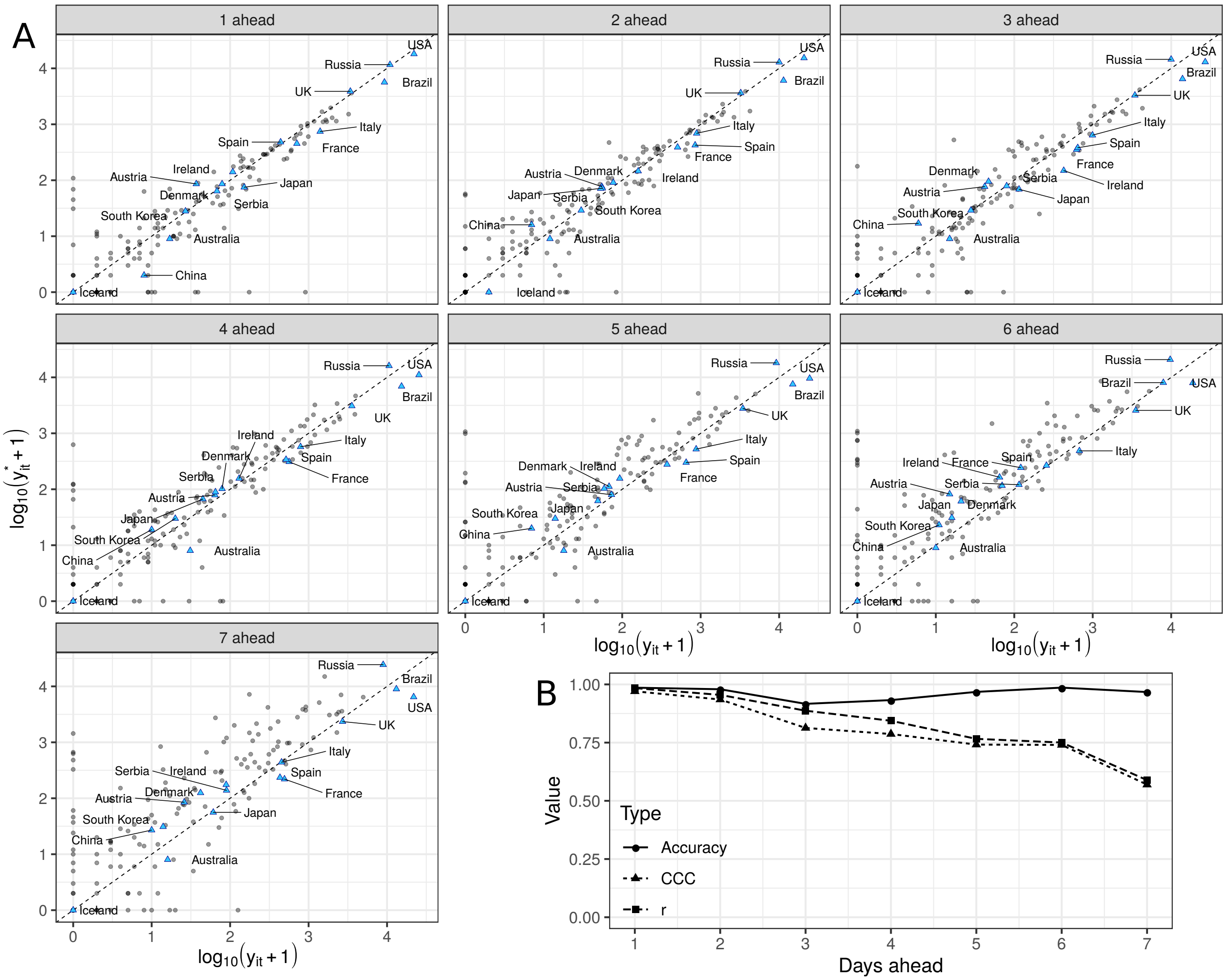

Figure S3. **A** Logarithm of the observed y_{it} versus the forecasted daily number of cases y^*_{it} for each country, for up to seven days ahead, where each day ahead constitutes one panel. The forecasts were obtained from the autoregressive state-space hierarchical negative binomial model, fitted using data up to 13-May-2020. The first day ahead corresponds to 14-May-2020, and the seventh to 20-May-2020. Each dot represents a country, and the sixteen countries shown in Figure 2 are represented by blue triangles. We add 1 to the values before taking the logarithm. **B** Observed accuracy, concordance correlation coefficient (CCC) and Pearson correlation (r) between observed (y_{it}) and forecasted (y^*_{it}) values for each of the days ahead of 13-May-2020.

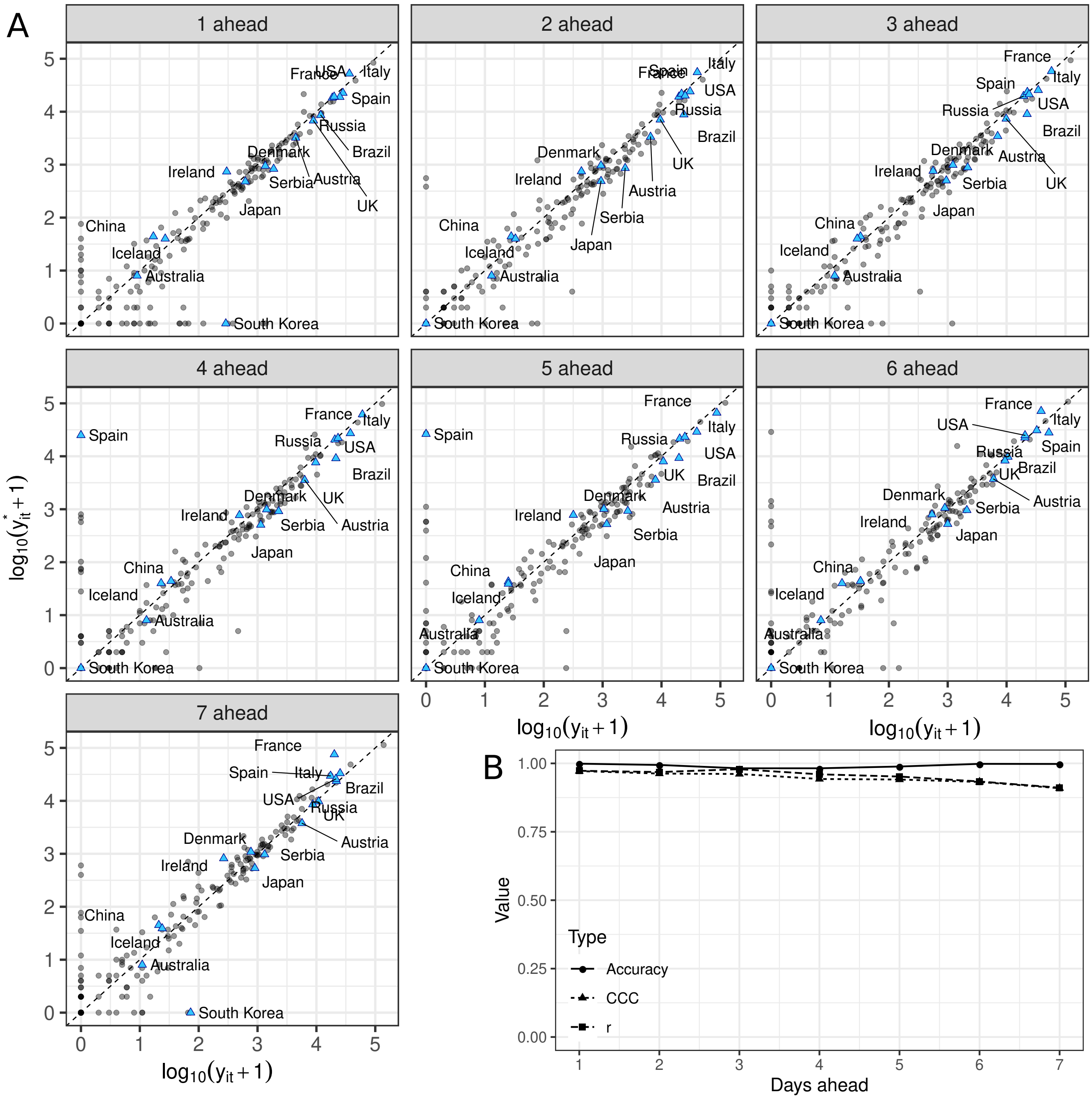

Figure S4. **A** Logarithm of the observed y_{it} versus the forecasted daily number of cases y^*_{it} for each country, for up to seven days ahead, where each day ahead constitutes one panel. The forecasts were obtained from the autoregressive state-space hierarchical negative binomial model, fitted using data up to 11-November-2020. The first day ahead corresponds to 12-November-2020, and the seventh to 18-November-2020. Each dot represents a country, and the sixteen countries shown in Figure 2 are represented by blue triangles. We add 1 to the values before taking the logarithm. **B** Observed accuracy, concordance correlation coefficient (CCC) and Pearson correlation (r) between observed (y_{it}) and forecasted (y^*_{it}) values for each of the days ahead of 11-November-2020.

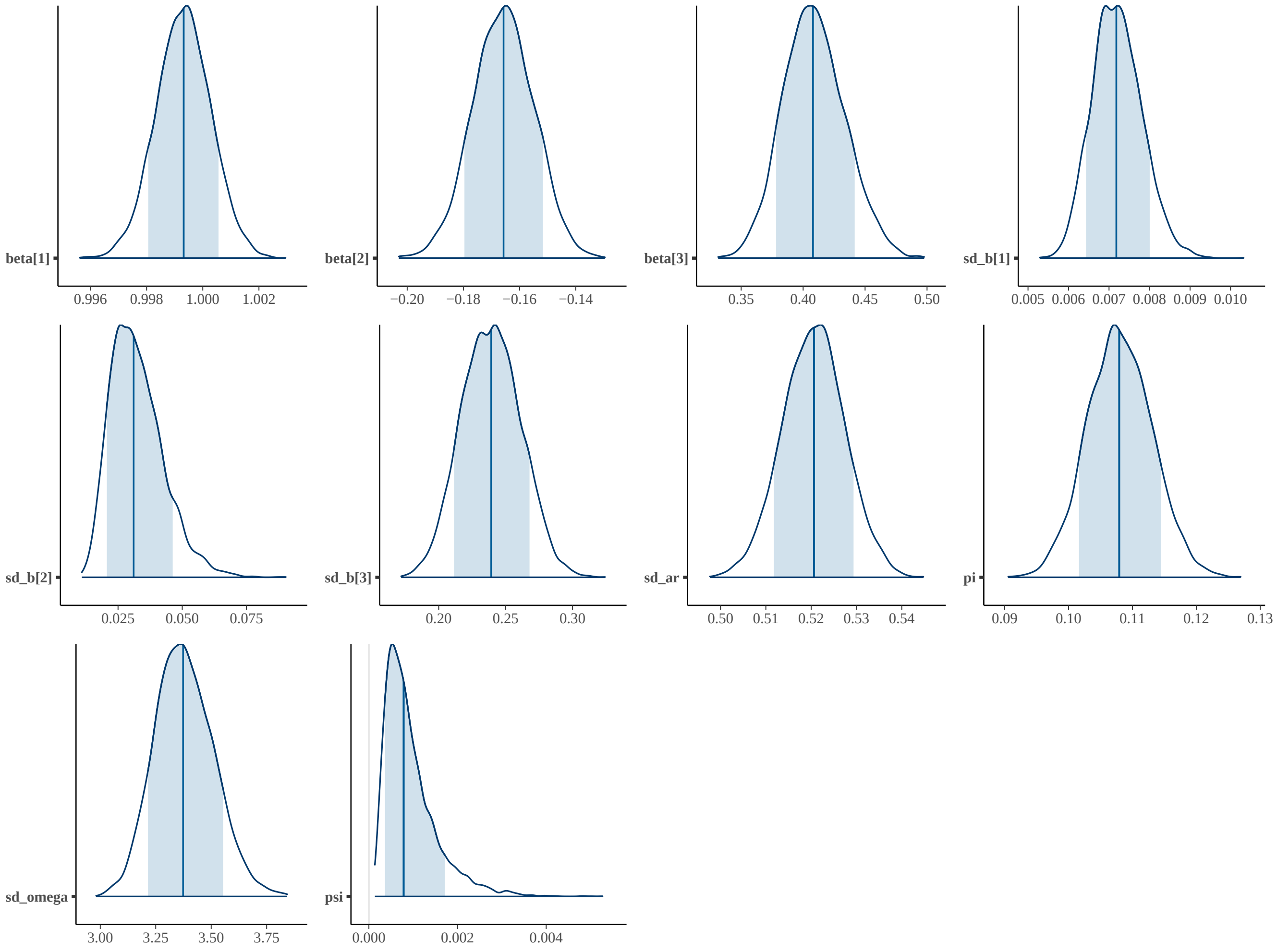

Figure S5. Density plots for the sampled posterior distributions for each model parameter.

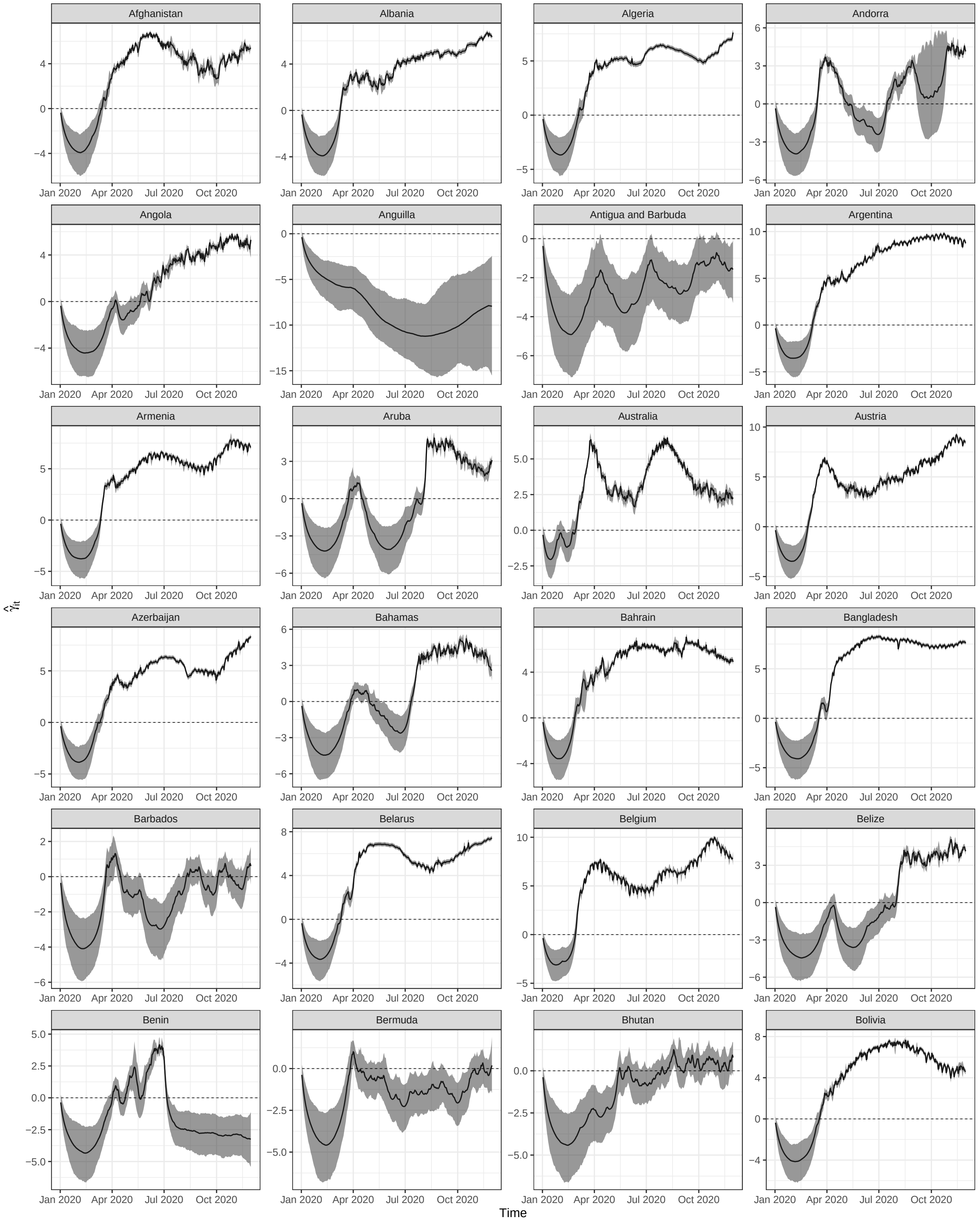

Figure S6. Posterior means of the autoregressive component γ_{it} (solid lines) and associated 95% credible intervals (shaded areas) for 24 countries from the pool of 214 countries and territories in the data, from 1-Jan-2020 until 25-Nov-2020.

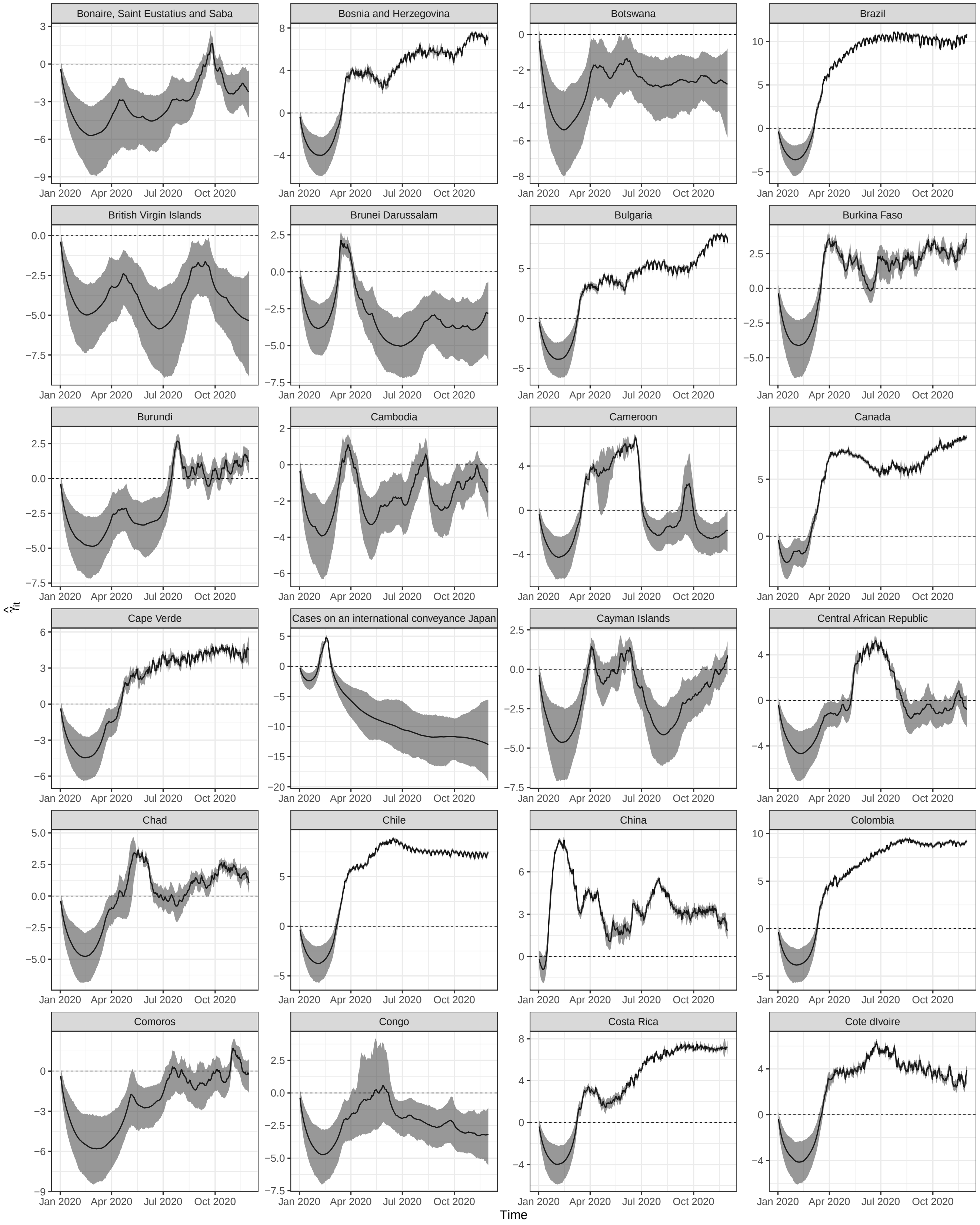

Figure S7. Posterior means of the autoregressive component γ_{it} (solid lines) and associated 95% credible intervals (shaded areas) for 24 countries from the pool of 214 countries and territories in the data, from 1-Jan-2020 until 25-Nov-2020.

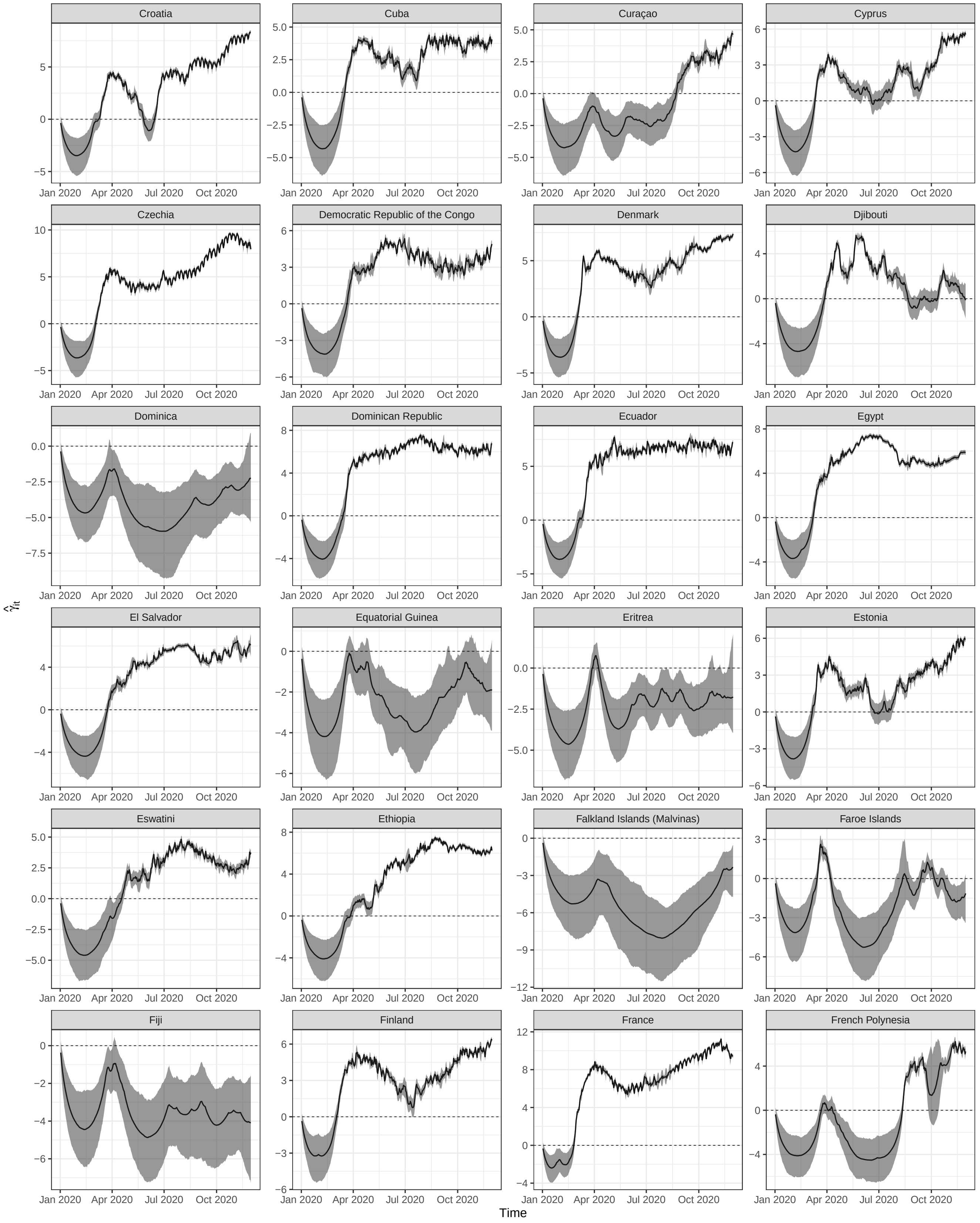

Figure S8. Posterior means of the autoregressive component γ_{it} (solid lines) and associated 95% credible intervals (shaded areas) for 24 countries from the pool of 214 countries and territories in the data, from 1-Jan-2020 until 25-Nov-2020.

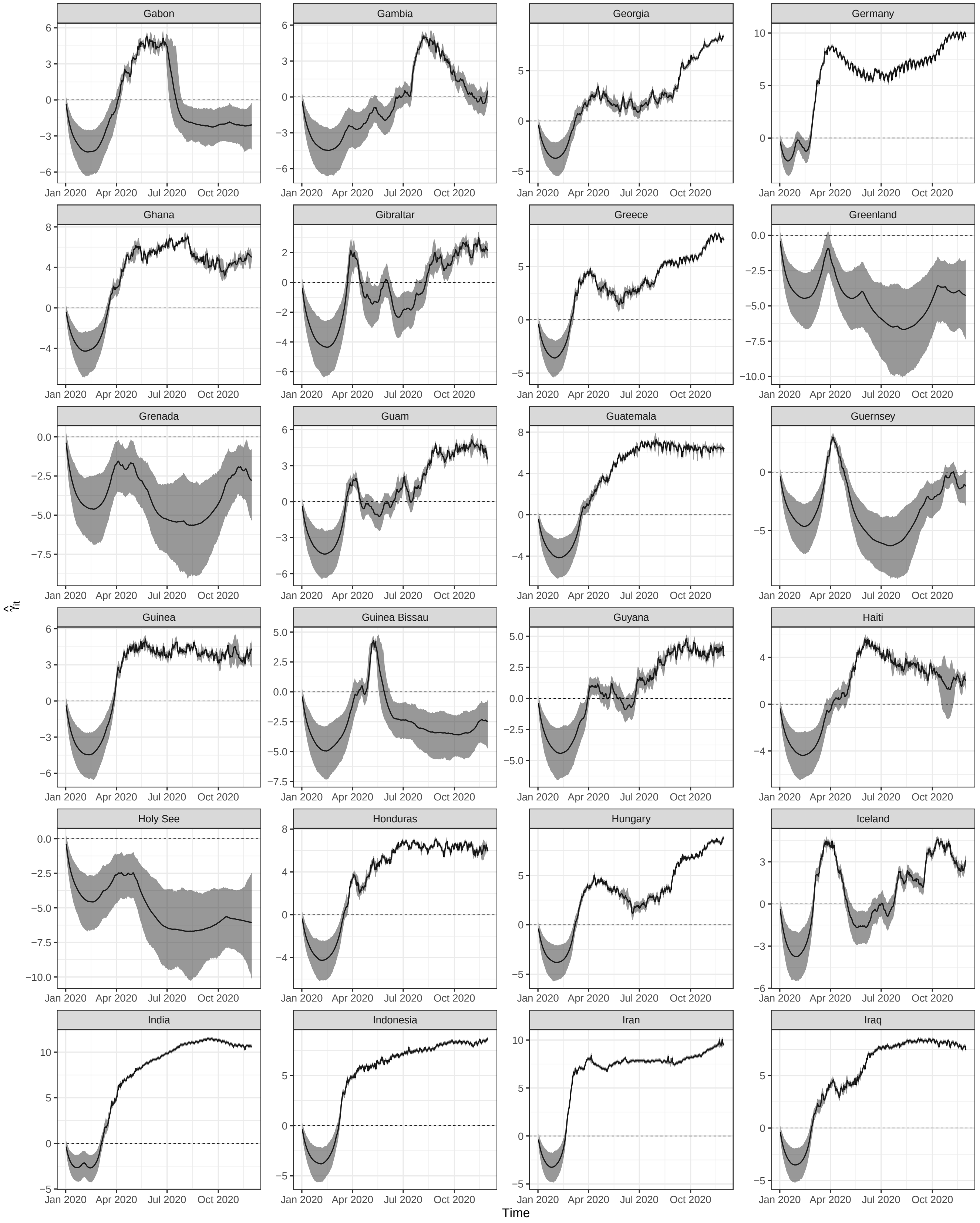

Figure S9. Posterior means of the autoregressive component γ_{it} (solid lines) and associated 95% credible intervals (shaded areas) for 24 countries from the pool of 214 countries and territories in the data, from 1-Jan-2020 until 25-Nov-2020.

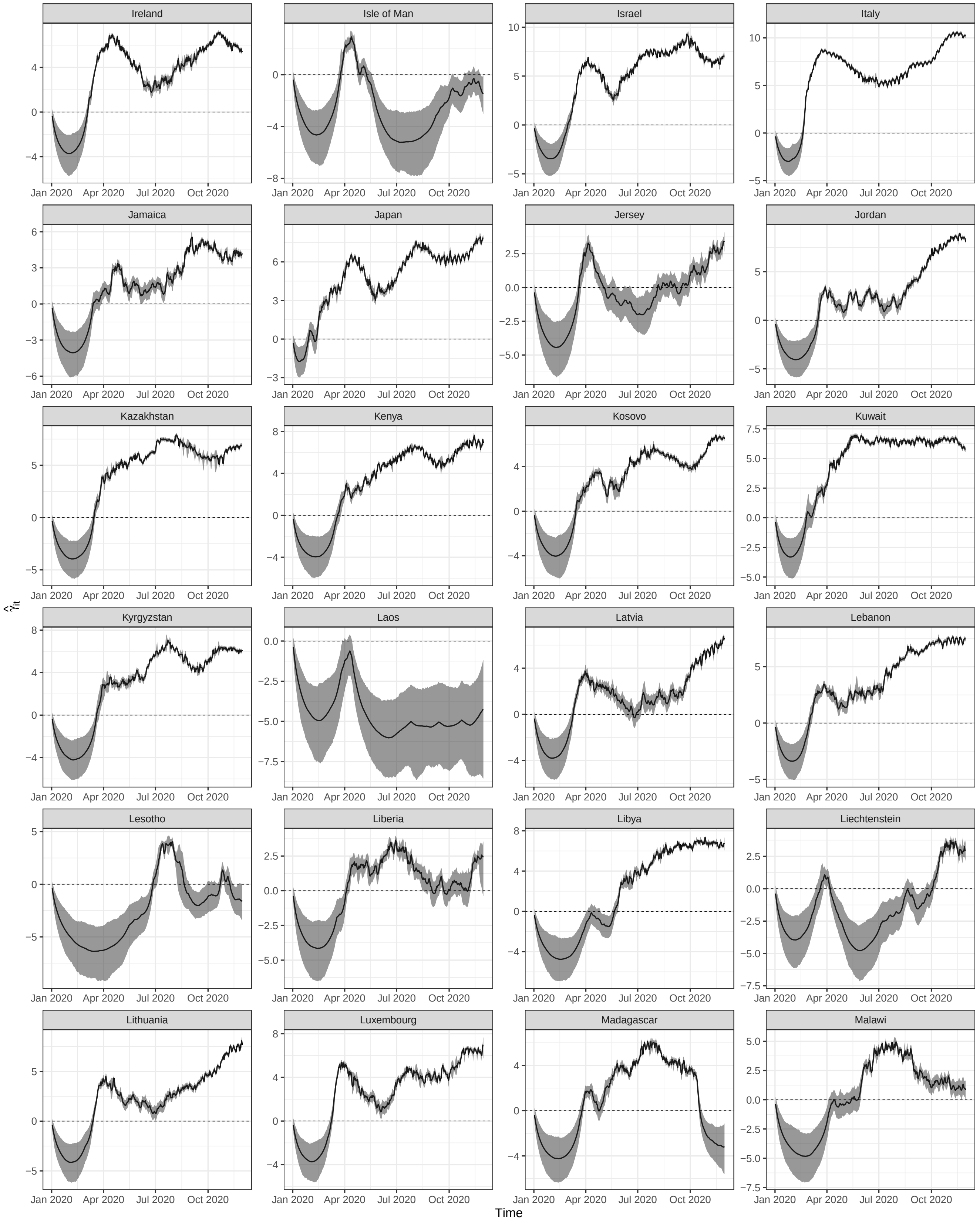

Figure S10. Posterior means of the autoregressive component γ_{it} (solid lines) and associated 95% credible intervals (shaded areas) for 24 countries from the pool of 214 countries and territories in the data, from 1-Jan-2020 until 25-Nov-2020.

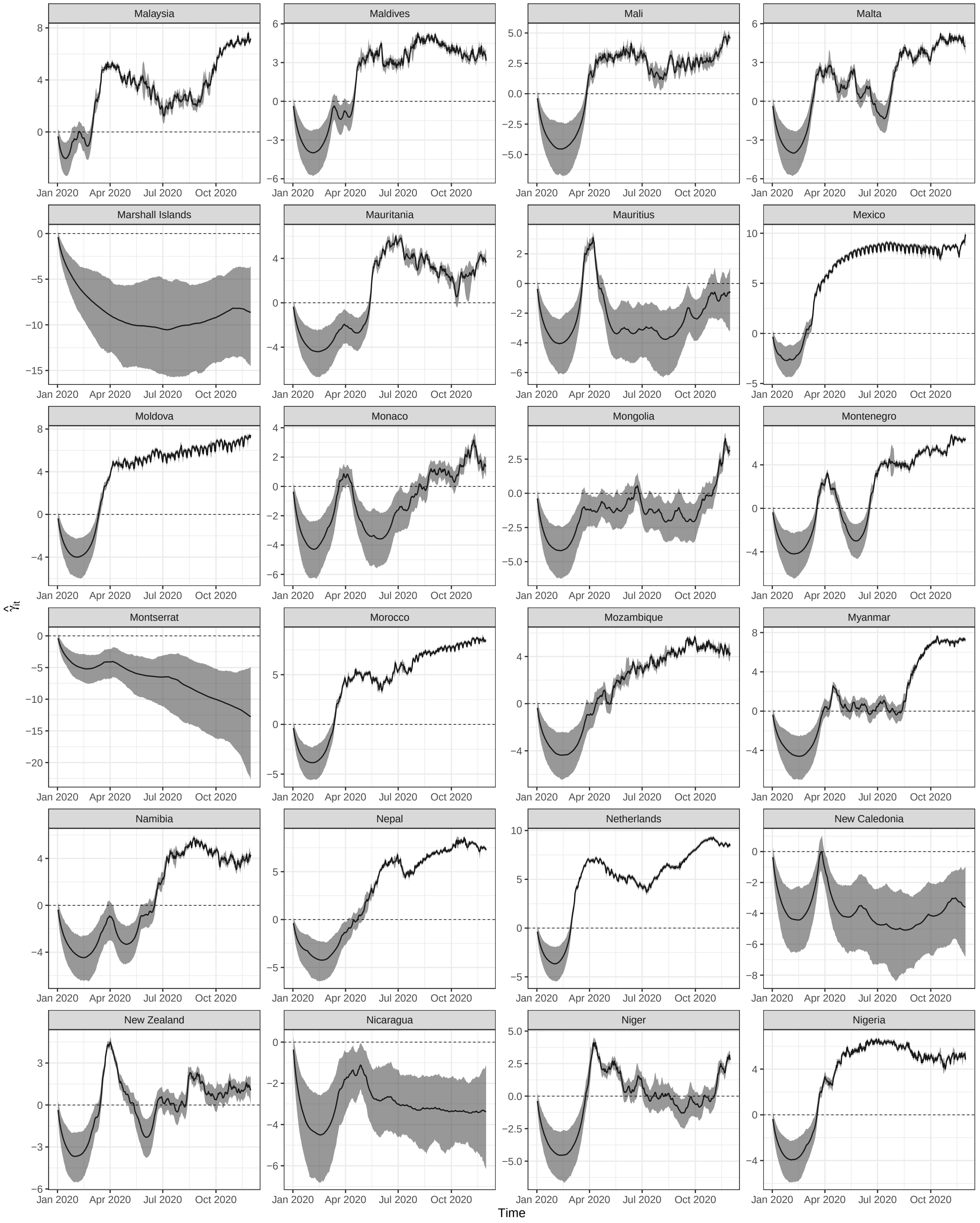

Figure S11. Posterior means of the autoregressive component γ_{it} (solid lines) and associated 95% credible intervals (shaded areas) for 24 countries from the pool of 214 countries and territories in the data, from 1-Jan-2020 until 25-Nov-2020.

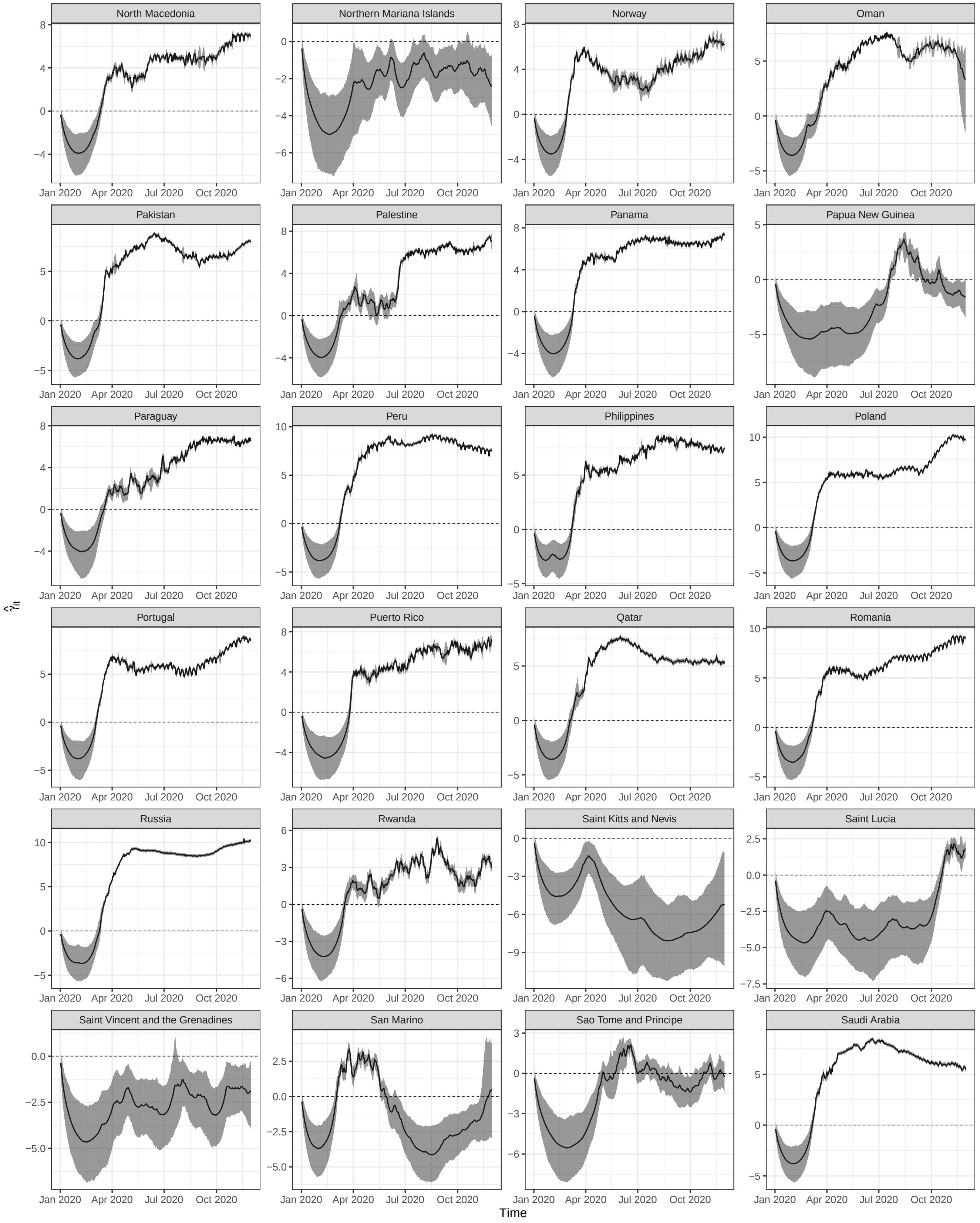

Figure S12. Posterior means of the autoregressive component γ_{it} (solid lines) and associated 95% credible intervals (shaded areas) for 24 countries from the pool of 214 countries and territories in the data, from 1-Jan-2020 until 25-Nov-2020.

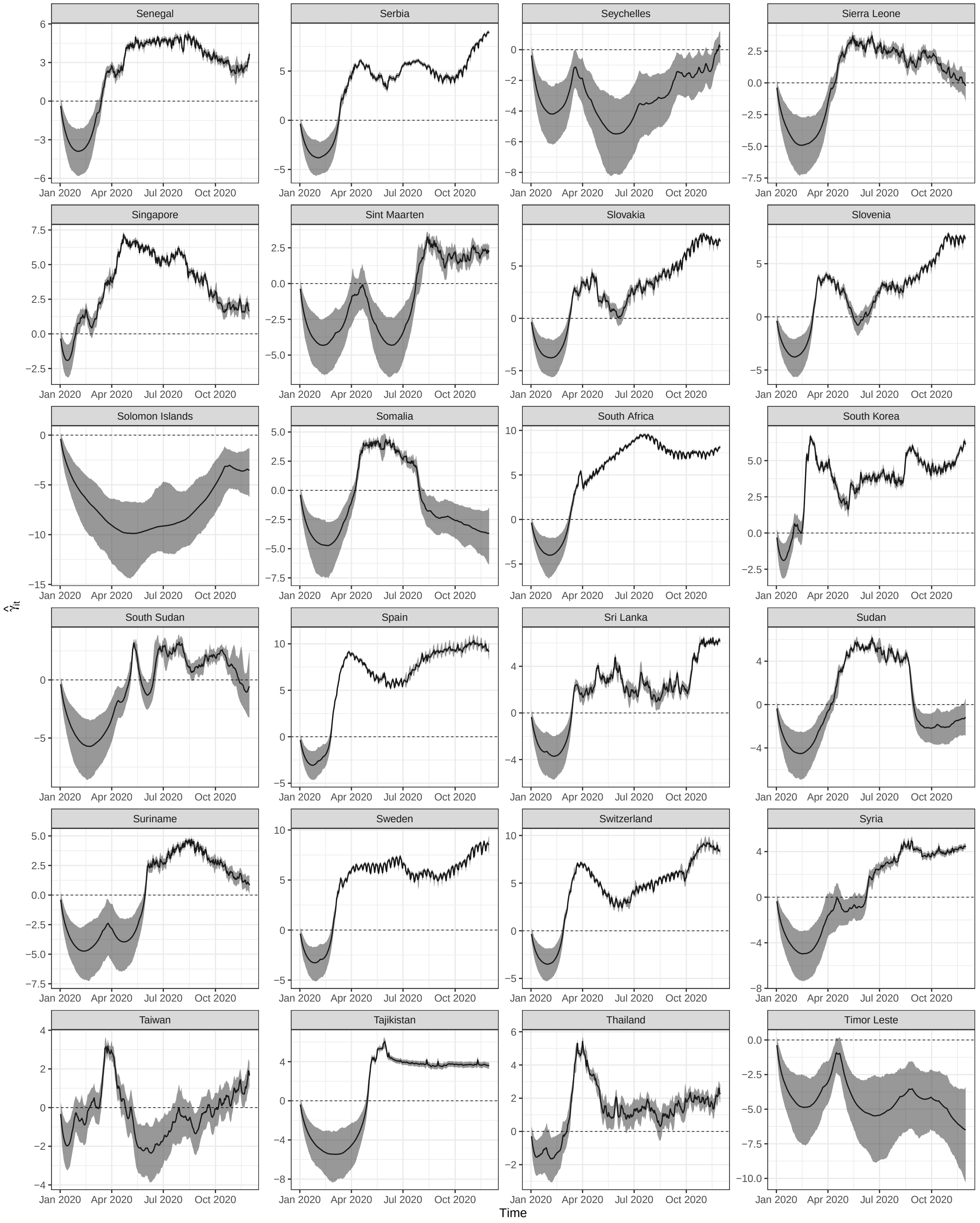

Figure S13. Posterior means of the autoregressive component γ_{it} (solid lines) and associated 95% credible intervals (shaded areas) for 24 countries from the pool of 214 countries and territories in the data, from 1-Jan-2020 until 25-Nov-2020.

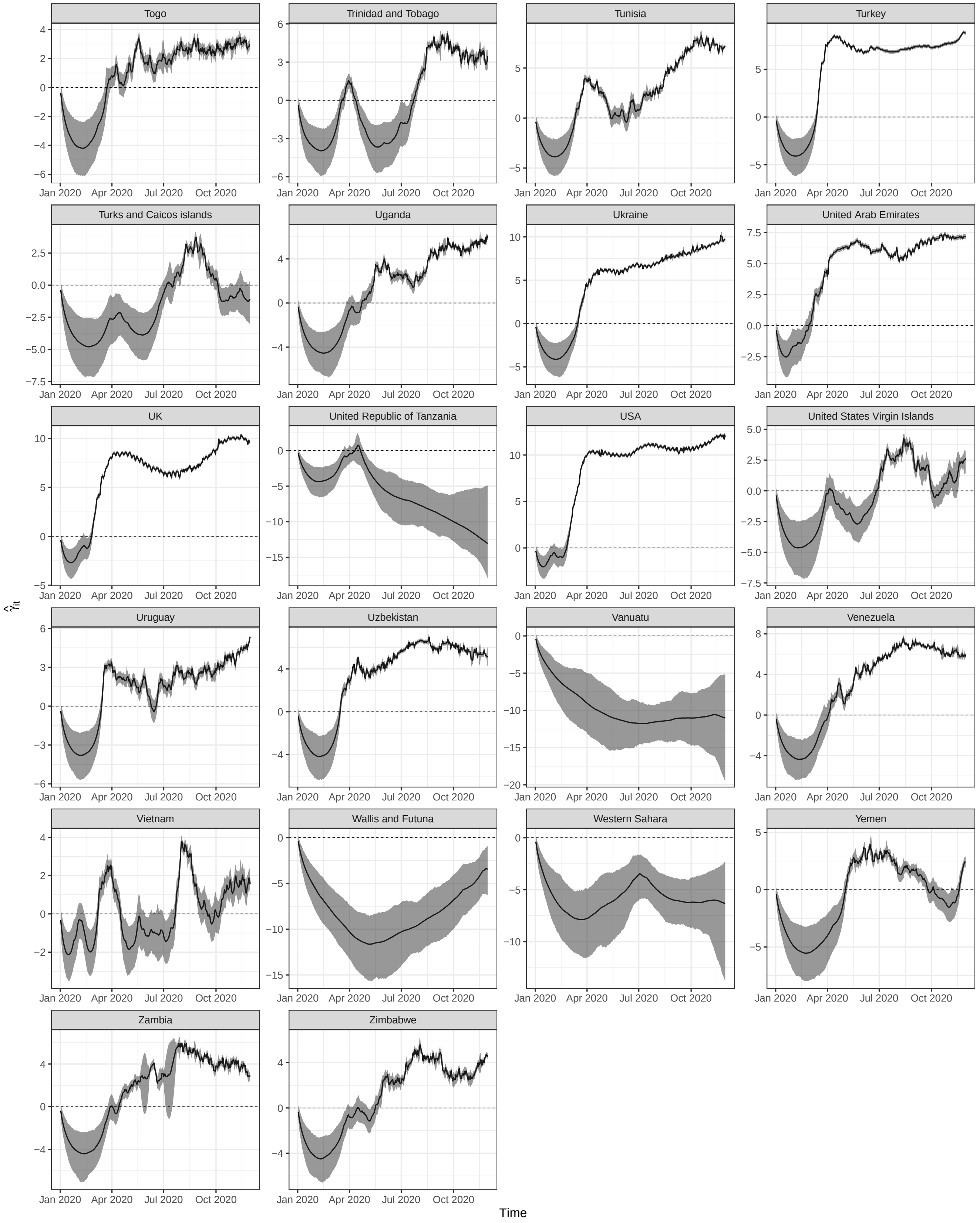

Figure S14. Posterior means of the autoregressive component γ_{it} (solid lines) and associated 95% credible intervals (shaded areas) for 22 countries from the pool of 214 countries and territories in the data, from 1-Jan-2020 until 25-Nov-2020.
